# Behavioral economic methods to inform infectious disease response: Prevention, testing, and vaccination in the COVID-19 pandemic

**DOI:** 10.1371/journal.pone.0258828

**Published:** 2022-01-19

**Authors:** Justin C. Strickland, Derek D. Reed, Steven R. Hursh, Lindsay P. Schwartz, Rachel N. S. Foster, Brett W. Gelino, Robert S. LeComte, Fernanda S. Oda, Allyson R. Salzer, Tadd D. Schneider, Lauren Dayton, Carl Latkin, Matthew W. Johnson

**Affiliations:** 1 Department of Psychiatry and Behavioral Sciences, Johns Hopkins University School of Medicine, Baltimore, MD, United States of America; 2 Department of Applied Behavioral Science, University of Kansas, Lawrence, KS, United States of America; 3 Cofrin Logan Center for Addiction Research and Treatment, University of Kansas, Lawrence, KS, United States of America; 4 Applied Behavioral Biology Unit, Institutes for Behavior Resources, Baltimore, MD, United States of America; 5 Department of Health, Behavior and Society, Johns Hopkins University Bloomberg School of Public Health, Baltimore, MD, United States of America; University of Edinburgh, UNITED KINGDOM

## Abstract

The role of human behavior to thwart transmission of infectious diseases like COVID-19 is evident. Psychological and behavioral science are key areas to understand decision-making processes underlying engagement in preventive health behaviors. Here we adapt well validated methods from behavioral economic discounting and demand frameworks to evaluate variables (e.g., delay, cost, probability) known to impact health behavior engagement. We examine the contribution of these mechanisms within a broader response class of behaviors reflecting adherence to public health recommendations made during the COVID-19 pandemic. Four crowdsourced samples (total N = 1,366) completed individual experiments probing a response class including social (physical) distancing, facemask wearing, COVID-19 testing, and COVID-19 vaccination. We also measure the extent to which choice architecture manipulations (e.g., framing, opt-in/opt-out) may promote (or discourage) behavior engagement. We find that people are more likely to socially distance when specified activities are framed as high risk, that facemask use during social interaction decreases systematically with greater social relationship, that describing delay until testing (rather than delay until results) increases testing likelihood, and that framing vaccine safety in a positive valence improves vaccine acceptance. These findings collectively emphasize the flexibility of methods from diverse areas of behavioral science for informing public health crisis management.

## Introduction

The COVID-19 pandemic has spurred important conversations in nearly all sciences [1–3]. While scientists scramble to find ways to address the crisis, a salient role for behavioral science has emerged. Robust evidence documents how human behavior is central to disease spread in so far as reduced travel and physical distancing (also known as social distancing) are predictive of lower infection incidence [4, 5] and facemask use effectively mitigates airborne transmission [6, 7]. As of this writing, only remdesivir has been approved by the FDA as an approved safe and effective pharmaceutical for COVID-19 treatment. Importantly, remdesivir and other therapeutics are designed for the treatment of existing COVID-19 symptoms rather than for prophylactic prevention, leaving nonpharmaceutical interventions as critical for flattening the curve of transmission [8]. Furthermore, while effective vaccines have been developed and subsequently approved and disseminated, behavioral science remains necessary for ensuring necessary vaccination rates by informing the development of public health programs to counteract factors like vaccine mistrust, skepticism, and apathy [9, 10]. Theoretical commentaries have further emphasized this role of understanding human behavior to thwart the transmission of infectious diseases like COVID-19 [11].

Behavioral economics provides a cogent and widely used framework for understanding a broad response class of behaviors relevant to preventive health and adherence to public health guidance. Behavioral economics may be defined as an approach to understanding behavior and decision making that integrates behavioral science (commonly psychology) with economic principles [12]. Research in this tradition has applied economic principles to understand decision making using methods often developed within operant psychology frameworks (e.g., purchasing of competing goods from an economist’s perspective may be the division of operant behavior among competing reinforcers from a behavioral psychologist’s perspective). This approach involves evaluation of behavioral mechanisms including delay discounting (i.e., the devaluation of an outcome by delay), probability discounting (i.e., the devaluation of an outcome by probability/certainty), and behavioral economic demand (i.e., relationship price and consumption that considers this relation may differ across people and contexts) to determine how these measurable factors influence choice and behavioral allocation as well as individual difference variables impacting these relationships. Studies conducted in this tradition provide additional applied benefits for human health and decision-making by informing interventions to encourage or “nudge” consumer choice toward socially desirable behaviors (e.g., through option framing), an approach commonly referred to as “choice architecture” [13].

The unique lens by which behavioral economics is used to describe behavior not only provides novel means of interpreting socially important concerns, but also the various facets of the dependent variables generated in these experiments render them especially useful for informing translational public policy [14–17]. For example, operant arrangements can quantify the effective price at which demand for a commodity shifts from inelastic (when a one unit increase in price is met with less than one unit decrease in consumption) to elastic (when a one unit increase in price is met with more than one unit decrease in consumption) for the consumer, while also modeling expected revenue on the part of the supplier. Moreover, comparisons of demand metrics have the potential to determine how imposing different environmental contexts (e.g., availability of reinforcer substitutes, closed economics, framing effects) alters the basic reinforcing value of the commodity for an individual as well as individual factors predictive of that influence. Similarly, discounting procedures can identify the effective delay [18] or probability [19] at which behavior is altered to a given level of performance; for example, the delay associated with, say, a 50% reduction in the value of procuring a COVID-19 test. Such metrics are ripe for modeling policy effects and can provide novel and important behavioral information that is directly relatable to the design of behavior change programs [16, 20, 21].

As described in recent commentaries [22–26], the mechanisms and methods described by behavioral economics may help inform decisions underlying COVID-19 preventive health behaviors and adherence to prescribed public health guidance. Empirically, research conducted in Italy during early lockdown (29 March to 4 April 2020) found that hypothetical engagement in compliance measures decreased with the expected time of compliance (e.g., less compliance at 180 days from now than today) and that this decrease was well described by a hyperbolic-like curve [27]. That study also found that a frame describing the hypothetical risk of contracting COVID-19 influenced the discounting of compliance by delay with greater discounting observed under low-risk conditions compared to medium- or high-risk ones. Similarly, our research group recently showed that describing the COVID-19 vaccine development process using a “rapid development” frame (modeled after the “Operation Warp Speed” initiative in the US) reduced vaccine acceptance in a behavioral economic demand task [28]. Decades of public health research have similar shown that validated behavioral economic methods adapted to assess health and policy-related contingencies (e.g., substance use, condom purchase and use) can be valuable tools to examine difficult-to-research scenarios. Importantly, when applied under hypothetical arrangements that simulate real-world decision-making, these behavioral economic demand and discounting procedures are significantly related to or predictive of actual consumption [29–32] or clinical measures associated with risky behaviors [33–36].

In this series of experiments, we sought to extend this work by applying behavioral economic methods and theory to understand public health response to the COVID-19 pandemic. This work is positioned within the broader conceptual framework of behavioral economic research reviewed above that demonstrates the impact of behavioral economic variables (e.g., delay, cost, probability, framing) on engagement with health behaviors such as substance use or preventive sexual health (e.g., condom use) [31, 33, 35, 37–40]. Here, we manipulate these behavioral economic dimensions by adapting them for direct relevance to the COVID-19 pandemic (e.g., the delay to receiving a COVID-19 testing result or cost of that test). We study the impact of these behavioral economic manipulations on a variety of behaviors related to COVID-19 prevention–physical (social) distancing, mask wearing, testing, and vaccination. Taken together, these findings demonstrate how manipulations of well-described behavioral economic variables adapted for novel contexts can offer insights into a broad response class underlying adherence to public health recommendations aimed to reduce infectious disease transmission.

## General methods

### Sampling and study overview

This manuscript summarizes a programmatic series of seven experiments conducted across four samples recruited asynchronously from March to September 2020 during the COVID-19 pandemic (see Table 1 for Sample overview). All samples were recruited using crowdsourcing (Amazon Mechanical Turk) with checks used to verify fidelity of responding. One experiment was formally pre-registered (Experiment 7; https://osf.io/56f2z) while others followed standard analyses based on the experimental design. Methods and Results are presented thematically based on the health behavior studied (i.e., physical distancing, facemask use, diagnostic testing, and vaccine intention).

**Table 1:** Sample overview.

				Demographics (Mean [SD]/%)			
Sample	Dates Sampled	Sampled N	Analyzed N	Age	Female	White	Experiments
1	3/13/20 to 3/17/20	227	133	39.5 (12.1)	59.8%	80.6%	1, 4
2	5/13/20 to 6/10/20	499	414	32.6 (10.9)	58.3%	71.5%	5
3	8/4/20 to 8/12/20	531	497	40.0 (11.4)	56.9%	78.7%	2, 3, 6
4	9/12/20 to 9/23/20	485	322	38.8 (11.6)	44.5%	76.7%	7

Note. Experiment reflects sample from which data reported here were collected.

Across all studies, we required participants to be age 18 or older and have United States residence. Additional attention and validity checks were included for each sample. A detailed summary of recruitment methods is available in S1 Text. All studies were reviewed and approved by local Institutional Review Boards (University of Kansas or Johns Hopkins University). Participants reviewed a study cover letter to provide electronic informed consent prior to participation.

### Experiment methods and data analysis

All experimental materials (e.g., experimental vignettes) are available in the S2 Text. Data were collected via Qualtrics and analyses conducted in *R* (see https://osf.io/wdnmx/?view_only=37323f9431aa4c91a0e7209054058dbe for limited datasets and code for primary analyses).

## Physical distancing

Physical distancing is a first-line prevention strategy for reducing COVID-19 transmission. As described in the Introduction, existing evidence highlights how variations in described risk can influence decisions to engage in a physical distancing mitigation strategies [27]. In Experiments 1 and 2 we manipulate the behavioral economic independent variable of probability as probabilistic community COVID-19 risk to test its impact on the dependent variable of engagement in physical distancing guidelines. In Experiment 1, participants completed a probability discounting task evaluating the likelihood of attending a large social gathering given varying probabilities of community risk for a hypothetical disease under different symptom framing conditions. In Experiment 2, participants completed a probability discounting procedure evaluating the likelihood of engaging in a social activity based on community COVID-19 risk under different risk framing conditions based on a widely disseminated risk assessment infographic from the Texas Medical Association [41].

### Experiment 1 methods

Experiment 1 manipulated behavioral economic probability through probabilistic community symptom risk of a hypothetical illness. The primary dependent variable was likelihood of attending a social event, which belongs to the broader response class of adherence to physical distancing recommendations for physical distancing. Thus, Experiment 1 measured how the value of social interaction/social events was devalued by the probability of community disease symptom risk.

Participants completed a probability discounting task to evaluate likelihood of attending a large social gathering given the probability of disease risk in the community. The study vignette described a situation involving planned attendance at a large social event. The task included two experimental manipulations related to the symptoms’ description. First, the *symptom type* comprised a within-subject manipulation. A “Mild” version of the task described symptoms including dry cough, fatigue, fever, shortness of breath, and headache. A “Severe” version of the task included these symptoms in addition to difficulty breathing (requiring a medical ventilator). All participants completed these two task manipulations with a randomized order of completion. Second, the *symptom framing* was a between-subject manipulation. Half of participants randomly assigned to the “Label” condition (n = 69) saw the two tasks with a corresponding label for the symptom severity (e.g., “this group of symptoms is classified as mild”; “this group of symptoms is classified as severe”). The other half of participants were assigned to a “No Label” condition (n = 64) and were provided the same symptom lists with no designation of severity for the group of symptoms. This labeling manipulation was designed to determine the extent to which framing symptoms based on severity could alter discounting of probabilistic risk. This question was informed by the personal observation of widespread symptom framing evident in news reports at the time of the experiment and prior literature demonstrating framing effects relevant to disease symptoms and preventative behaviors [42]. Participants rated their likelihood of attending the social event at varying probabilities that someone in their community was presenting the symptoms described. Participants emitted responses on a visual analog scale (VAS) from 0 (extremely unlikely to attend) to 100 (extremely likely to attend). Symptom probabilities included 0%, 5%, 10%, 25%, 50%, 75%, 90%, 95%, and 100%.

Group discounting data were analyzed and plotted for descriptive purposes using the hyperbolic discounting equation that includes a non-linear scaling parameter [43]. Symptom probability was included in analyses as odds in favor of a symptomatic attendee. Individual discounting data were analyzed as area under the curve (AUC) to provide a model free estimation of the impact of symptom probability on the discounting of event attendance [44]. Lower AUC values indicate greater sensitivity to risk (the desirable outcome from a physical distancing standpoint). We standardized responses to the 0% likelihood response to isolate the impact of symptom probability from expected attendance at no-risk. We used an ordinal variation of AUC, here and throughout, to address concerns with normality and disproportional influence of delay or probability steps [45]. AUC values were analyzed using a 2 x 2 mixed ANOVA with the between-subject factor of Label (No Label versus Label) and within-subject factor of Symptom Type (Mild versus Severe). Generalized linear mixed effect models were used to test the likelihood of 100% likelihood of attendance at 0% community transmission risk (a bimodal distribution was observed; therefore, this outcome was dichotomized for analysis).

### Experiment 1 results: Framing effects of symptom severity for a hypothetical disease

**Fig 1:**
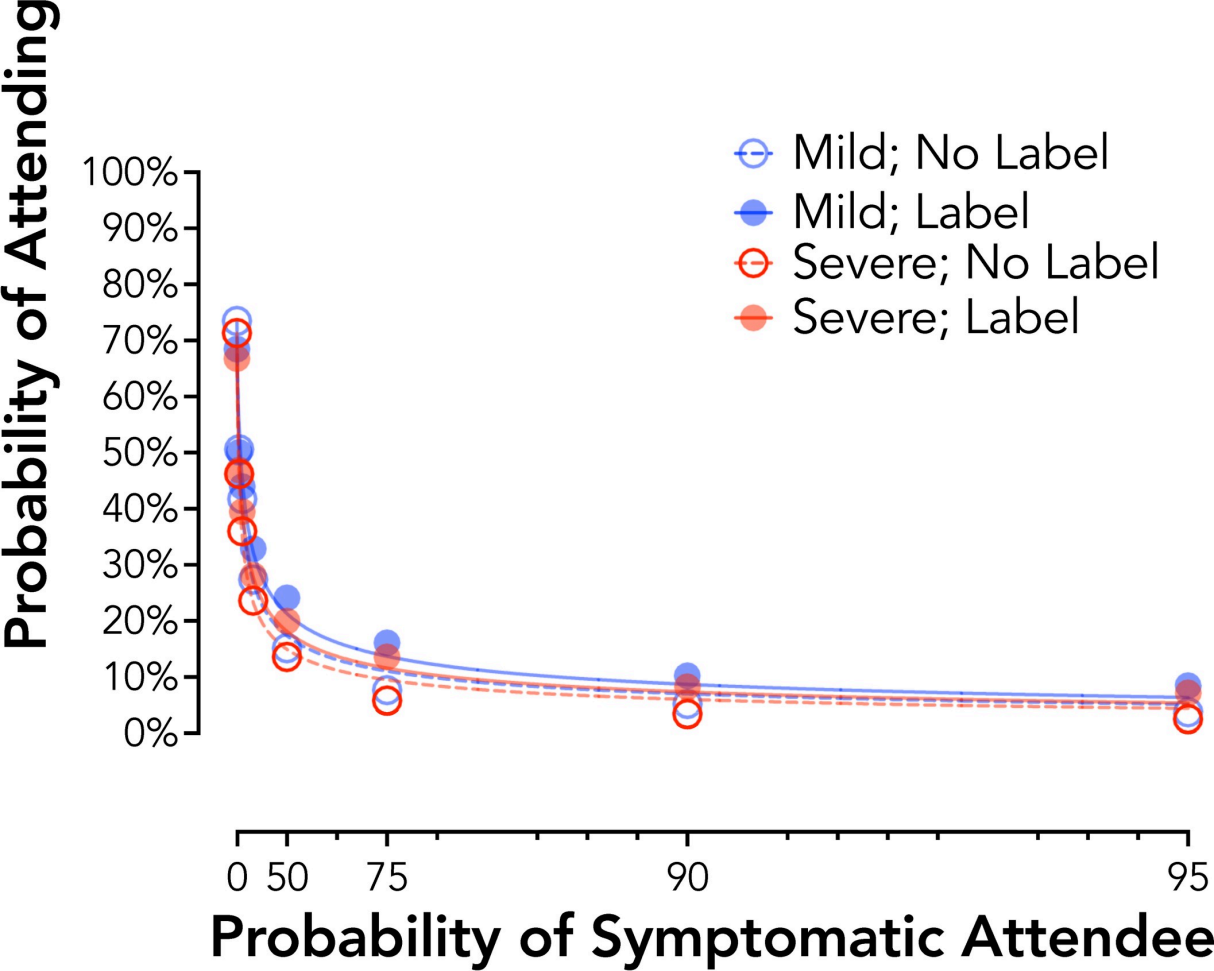
Probability discounting of social event attendance by symptom framing (Experiment 1). Plotted are group discounting curves by severity (mild = blue circles; severe = red circles) and label type (no label = open circles, dotted line; label = closed circles, solid line). X axis values are analyzed as odds in favor of a symptomatic attendee. Curves are plotted using the hyperbolic discounting equation including a non-linear scaling parameter [43].

Responding at an aggregate level showed systematic and expected decreases in attendance likelihood (i.e., increases in physical distancing) based on community symptom risk, mild no label *R*^*2*^ = .99, mild label *R*^*2*^ = .99, severe no label *R*^*2*^ = .99, severe label *R*^*2*^ = .99 (Fig 1). Individual AUC values revealed a significant main effect of Symptom Type, *F*_*1*_,_*131*_ = 12.75, *p* < .001, reflecting higher AUC values for the Mild than Severe symptoms, *d*_*z*_ = 0.31. The main effect of Label, *F*_*1*_,_*131*_ = 2.87, *p* = .09, and the Symptom Type by Label interaction, *F*_*1*_,_*131*_ = 0.01, *p* = .91, were not statistically significant. Generalized linear mixed effect models testing the likelihood of attendance at 0% community risk also showed no significant differences by Label or Symptom Type, *p* values > .09.

### Experiment 2 methods

Experiment 2 manipulated behavioral economic probability through probabilistic community symptom risk of COVID-19. The primary dependent variable was likelihood of engaging in a (personalized) social activity, which like Experiment 1 belongs to the broader response class of adherence to physical distancing recommendations. Thus, Experiment 2 measured how the value of a personalized social activity was devalued by the probability of community COVID-19 risk.

Similar to Experiment 1, participants completed a probability discounting task to evaluate likelihood of engaging in a social activity based on community COVID-19 risk. Participants were first asked to select a preferred social activity from a low-to-moderate risk category (i.e., play golf with others, go to a library or museum, or walk in a busy downtown) and from a high-risk category (i.e., go to a sports stadium, go to a movie theater, or attend a religious service with 500+ other worshipers). These groupings were based on the risk categorizations made by the Texas Medical Association in June 2020 [41]. Participants then read a vignette describing the opportunity to engage in that activity. A *risk framing* manipulation (between-subject) varied risk categorization with half of participants (N = 246) completing task that included labels for the risk severity (e.g., “According to health authorities in your area, this activity is of [Low/**High Risk]”)** and the other half of participants (N = 251) receiving no risk information. Tasks for the two selected activities were completed in a randomized order. Participants rated their likelihood of going to the social activity at varying probabilities that someone at the activity was displaying COVID-19 symptoms. Participants emitted responses on a VAS from 0 (definitely would not go) to 100 (definitely would go). Symptom probabilities included 0%, 1%, 5%, 10%, 25%, 50%, 75%, 99%, and 100%.

Group discounting data were analyzed and individual AUC values calculated as in Experiment 1. AUC values were analyzed using a 2 x 2 mixed ANOVA with the between-subject factor of risk Label (No Label versus Label) and within-subject factor of Risk Level (Low-to-Moderate versus High). Generalized linear mixed effect models were used to test the likelihood of 100% likelihood of attendance at 0% community transmission risk.

### Experiment 2 results: Framing effects of activity risk for COVID-19

**Fig 2:**
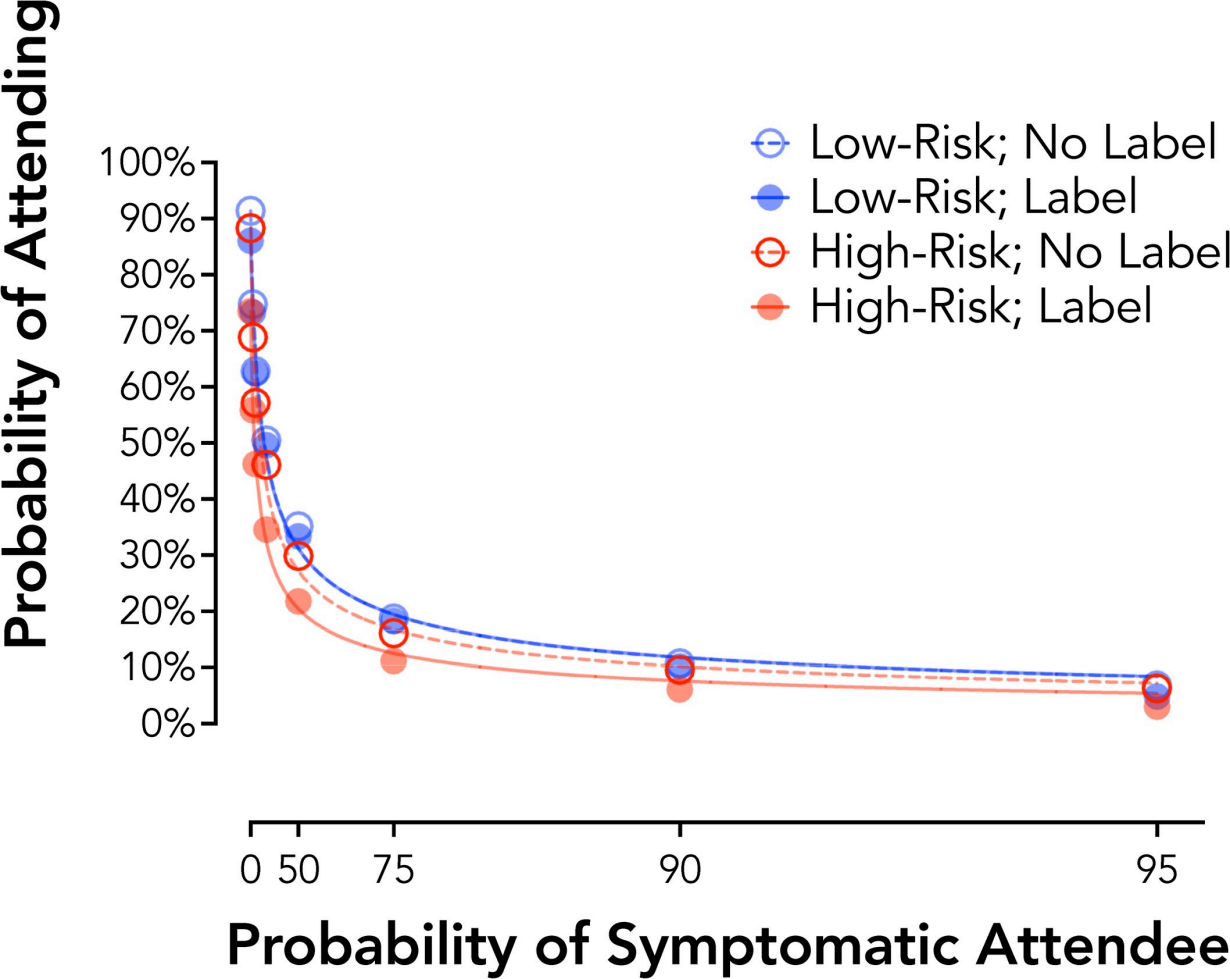
Probability discounting of social event attendance by risk framing (Experiment 2). Plotted are group discounting curves by risk (low risk activity = blue circles; high risk activity = red circles) and label type (no label = open circles, dotted line; label = closed circles, solid line). X axis values are computed as odds in favor of a symptomatic attendee. Curves are plotted using the hyperbolic discounting equation including a non-linear scaling parameter [43].

Responding at an aggregate level showed systematic and expected decreases in social activity likelihood (i.e., increases in physical distancing) based on community symptom risk as in Experiment 1, group curves low-risk no label *R*^*2*^ = .99, low-risk label *R*^*2*^ = .99, high-risk no label *R*^*2*^ = .99, high-risk label *R*^*2*^ = .99 (Fig 2). Standardized AUC values revealed a significant main effect of Risk Level, *F*_*1*_,_*495*_ = 113.62, *p* < .001, and a Risk Level by Risk Framing interaction, *F*_*1*_,_*495*_ = 26.47, *p* < .001. This interaction reflected no significant between-subject effect of Label for the Low Risk activity, *t*_495_ = 0.067, *p* = .95, *d* = -0.01, but in the Label group significantly lower AUC values (i.e., greater sensitivity to risk) for the High Risk activity than the Low Risk activity, *t*_495_ = 2.967, *p* = .003, *d* = -0.27. A significant Risk Level by Risk Framing interaction was also observed at the 0% probability of a symptomatic attendee, *b* = -2.59, *p* < .001. This interaction reflected no differences by Risk Framing in the likelihood of attendance at 0% risk for the Low Risk Activity, OR = 0.84, *p* = .40, but a lower likelihood of attendance for the High Risk Activity for the Label Risk Framing condition, OR = 0.48, *p* < .001.

### Physical distancing summary

These findings collectively show that physical distancing can be effectively modeled using probability discounting procedures. Across each experiment, adherence to physical distancing recommendations was measured through the dependent variable of engagement in social activities. The value of social interaction or activity was devalued by manipulation of the probability of both non-specific (Experiment 1) and COVID-19 specific (Experiment 2) community disease risk. The findings highlight a behavioral economic mechanism by which decisions to adhere to physical distancing guidelines are weighted, specifically against the probability of transmission risk (with physical distancing adherence greatest at the highest risk levels). Importantly, we found that a framing manipulation modeled after popular public health messaging targeting physical distancing increased sensitivity to probability of community transmission risk for a high-risk activity while not appreciable changing behavior for a low-risk activity. This is important given the potential for risk framing to have an untoward effect of reducing risk sensitivity for low-risk settings when presented in these kinds of behavioral contrasts (i.e., boomerang effects) [46].

## Facemask use

Consistent facemask use in social interactions is one of the most widely recommended means of reducing COVID-19 transmission [6, 7]. Despite its effectiveness, the use of facemasks remains controversial and underutilized [47]. Evaluating individual and contextual factors that influence facemask use may help to identify areas for intervention–either at a population-level (stemming from between-person differences) or at a contextual-level (stemming from within-person differences). In Experiment 3 we evaluated the role of social factors in determining facemask use. We specifically manipulate the behavioral economic independent variable of social distance (“social discounting”) to test its impact on the dependent variable of adherence to facemask guidelines. The notion that facemask use may relate to social context is reasonable given that one of the primary benefits of mask use is prevention of transmission to others. With respect to behavioral economic theory, social discounting is a well-described behavioral mechanism by which the value of an outcome is devalued by the “social distance” or subjective “closeness” of a person to the participant (e.g., a co-worker would be more socially distant than a sibling or parent). Empirical work on social discounting finds that the value of an outcome is hyperbolically devalued by social distance in a way that is mechanistically similar to delay and probability discounting [48–50]. Participants in Experiment 3 completed a traditional social discounting task using monetary consequences and a novel social discounting task with adherence to facemask use as the target behavior.

### Experiment 3 methods

Experiment 3 manipulated social discounting through a traditional social discounting framework (i.e., subjective closeness of a social partner). The primary dependent variable was likelihood of facemask use, which belongs to the broader response class of adherence to guidance in facemask use. Thus, Experiment 3 measured how the value of facemask use was devalued by the subjective closeness of a social partner.

Participants completed social discounting tasks evaluating responding for facemask as well as a task for monetary consequences [50]. Prior to completion of the social discounting tasks, participants were asked to think of the 100 people closest to them with 1 being the dearest friend or relative in the world and 100 being a mere acquaintance. Participants were then asked to record their relation to people at numbers 5, 10, 20, 50, and 100 on this list, all of which were people instructed to be someone they did not live with or see in the past month. This information was included in the response options to personalize responding. In the facemask version of the task, participants were instructed to report their likelihood of wearing a facemask when interacting with people at each of these social distances using a 100-point VAS. Three conditions were presented including 1) when the participant was COVID-19 asymptomatic, 2) COVID-19 symptomatic without a positive test, and 3) COVID-19 symptomatic with a positive test. These conditions were presented in that order to model the progression that a person may experience in decision-making (i.e., asymptomatic to positive). Participants also completed a social discounting task for money based on prior methods [48]. In this task, participants were asked to select between receiving an amount of money for themselves alone or $75 USD for the N person on the list. Participants completed this task for the 5, 10, 20, 50, and 100 on their social list. Participants were also asked about responding for a stranger in each task version (facemask and monetary tasks).

Group discounting data (N = 452) were analyzed as in Experiment 1. Generalized linear mixed effect models were used to test the likelihood of using a facemask across all social distances as a function of condition (asymptomatic, symptomatic no test, symptomatic test). Spearman correlations evaluated the association of responding on the asymptomatic facemask task (the task with the greatest variability) and social discounting task for monetary outcome.

### Experiment 3 results

**Fig 3:**
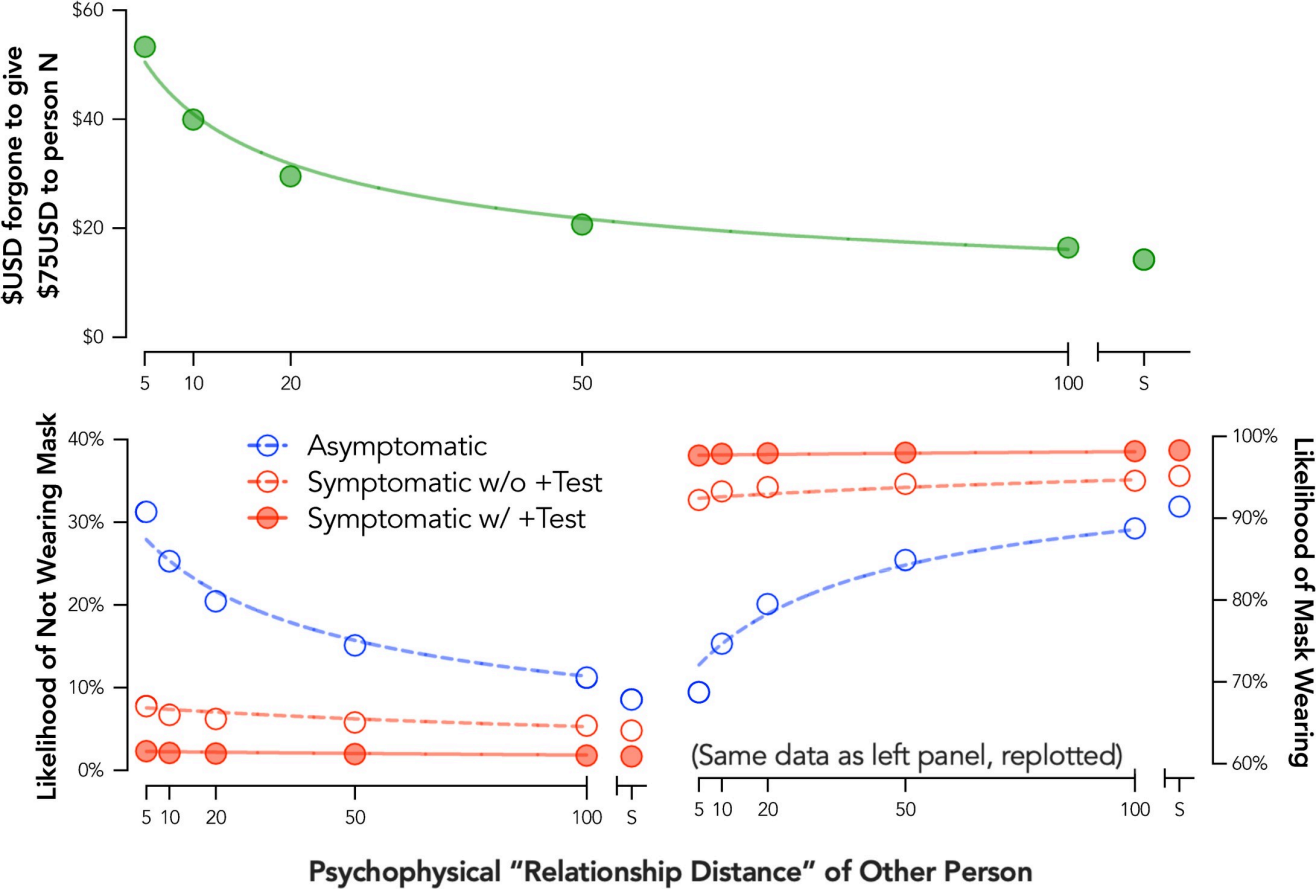
Social discounting for facemask use and monetary outcomes. Plotted are group discounting curves for money (top panel) and facemask use (bottom panels). Three facemask use conditions are presented: asymptomatic (red open circles, dotted line), was symptomatic without a COVID-19 test (red open circles, dotted line), and 3) was symptomatic with a positive COVID-19 test (red closed circles, solid line). Curves are plotted using the hyperbolic discounting equation including a non-linear scaling parameter [43]. S = stranger.

Prior to data collection, we expected that the likelihood of using a facemask would be devalued by social distance such that the greater the social distance, the lower likelihood of mask use. Surprisingly, an opposite and systematic pattern of behavior was observed–participants reported *greater* likelihood of facemask use with increasing social distance in an orderly fashion (Fig 3; bottom right). These data suggest a systematic discounting pattern, but that the devalued outcome was likely not facemask use. Instead, these findings suggested that the value of social interaction without a facemask was devalued by the subjective closeness of a social partner.

When recoding responses to measure likelihood of interaction without a facemask, a systematic discounting pattern was observed with the likelihood of interacting without a mask discounted hyperbolically by social distance, group curve *R*^*2*^ for asymptomatic responding = .96, symptomatic no test responding = .66, symptomatic with positive test responding = .71 (Fig 3; bottom left). Note that the relatively poor fit for the discounting functions in Experiment 3 for the two symptomatic conditions is likely related to the relative flatness of these two functions. As described previously [51], *R*^*2*^ values for a non-linear function like the hyperbolic discounting equation are biased towards reporting a better “fit” for higher rates of discounting.

Critically, responding on the monetary social discounting task followed an expected and typical pattern in which the amount of money foregone to a social partner was systematically and hyperbolically discounted by increasing social distance, group curve *R*^*2*^ = .98 (Fig 3 top panel). This finding suggests that the unexpected pattern of response on the facemask task was not due to issues with instructional control or participant understanding of the social distance manipulation. Social discounting for monetary and face mark outcomes were not significantly correlated, *r* = -.07, *p* = .18.

A clear effect of symptomatic status was also observed such that facemask use was more likely (i.e., likelihood of forging a mask was lower) when a participant was symptomatic whether with or without a positive test (Fig 3). This pattern of response was attributable, in part, to an increase in the proportion of participants reporting they would wear a mask when interacting with any social partner in the asymptomatic condition (44.9%) to the symptomatic condition (78.8%, OR = 32.1, *p* < .001) and positive test condition (94.0%, OR = 604.1, *p* < .001).

### Facemask use summary

These findings show that facemask use is sensitive to manipulation of the behavioral economic factor of social closeness within a social discounting framework. Our assumption based on existing social discounting research was that one would be more likely to wear a mask with those who are interpersonally close given concern about infecting those they care about. However, it appears that the increased value of mask-less interaction with those one is interpersonally closer to may overshadow this additional concern for that risk for transmission. Unsettlingly, these findings suggest that the value of mask-less social interaction is the more salient factor considered when deciding whether to use or not use a mask based on social relations to others. Relevant to note is that participants were asked to respond to these questions for people that they had not seen in the past month and did not live with. Therefore, these findings cannot be accounted for by responding based on people who they person has already had recent close and mask-less contact with. These data are also in line with other literature emphasizing the value of facial expression for emotional and social interaction [52, 53].

While social discounting is typically considered to measure generosity or altruism, at a more functional/foundational level, social discounting measures the degree to which the value of a consequence is devalued (discounted) by social distance from another person. The non-significant correlations with social discounting for monetary outcomes further suggest a decoupling of the impact of manipulating social distance on facemask use from an altruistic view of the health of others (and possibly overshadowing by the benefits or value of unimpeded social interactions). Future work would be beneficial to expand upon this preliminary, but novel finding, and determine methods to potentially mitigate this non-adherence to public health recommendations.

## Diagnostic testing

COVID-19 testing is central to identifying infection status to prevent future transmission as well as to inform contact tracing. However, difficulties in obtaining testing and subsequent delays related to receiving results were a noted criticism of COVID-19 efforts. Experiments 4 and 5 were designed to evaluate decision-making processes when considering obtaining a COVID-19 diagnostic test. In Experiment 4, we manipulate the behavioral economic independent variable of demand (price) to test its impact on decisions to purchase a diagnostic test following possible exposure to a hypothetical disease. In Experiment 5, we build on these findings to evaluate the behavioral economic variables of delay, price, and delay framing in decisions to obtain a COVID-19 diagnostic test.

### Experiment 4 methods

Experiment 4 manipulated price using a hypothetical purchase task procedure. The primary dependent variable was likelihood of purchasing a diagnostic test for a hypothetical disease, which is a behavior belonging to the broader response class of adherence to testing recommendations (i.e., getting tested after possible exposure and symptom expression). Thus, Experiment 4 measured how the value of a diagnostic test (and more broadly adherence to this guideline) decreased by the price of that test.

Participants completed a hypothetical purchase task procedure to evaluate behavioral economic demand for a diagnostic testing kit for a hypothetical disease. Specifically, participants read a vignette indicating that they had attended a social event with over 200 people and one week later developed symptoms including cough, fever, and shortness of breath. Participants were also instructed that one other person in their county had developed an infection, that a nearby hospital or clinic had a testing kit, but that there were no others in the area, that this kit was approved by the Centers for Disease Control and Prevention (CDC), and that they had their typical income and savings available when making these decisions. Participants were asked to report the likelihood of purchasing a testing kit given a series of out-of-pocket costs ($0 [free], $1, $5, $10, $20, $30, $40, $50, $75, $100, $150, $200, $500, $1,000, $2,000, and $5,000/kit). Participants emitted responses on a VAS from 0 (extremely unlikely to get tested) to 100 (extremely likely to get tested).

A group mean demand curve was fit using the exponential demand equation [54] to evaluate the point of unit elasticity (i.e., analytical P_max_ value) reflecting the point at which a one-log unit increase in price is met by a one log-unit decrease in consumption [55].

### Experiment 4 results: Sensitivity of diagnostic testing to cost

**Fig 4:**
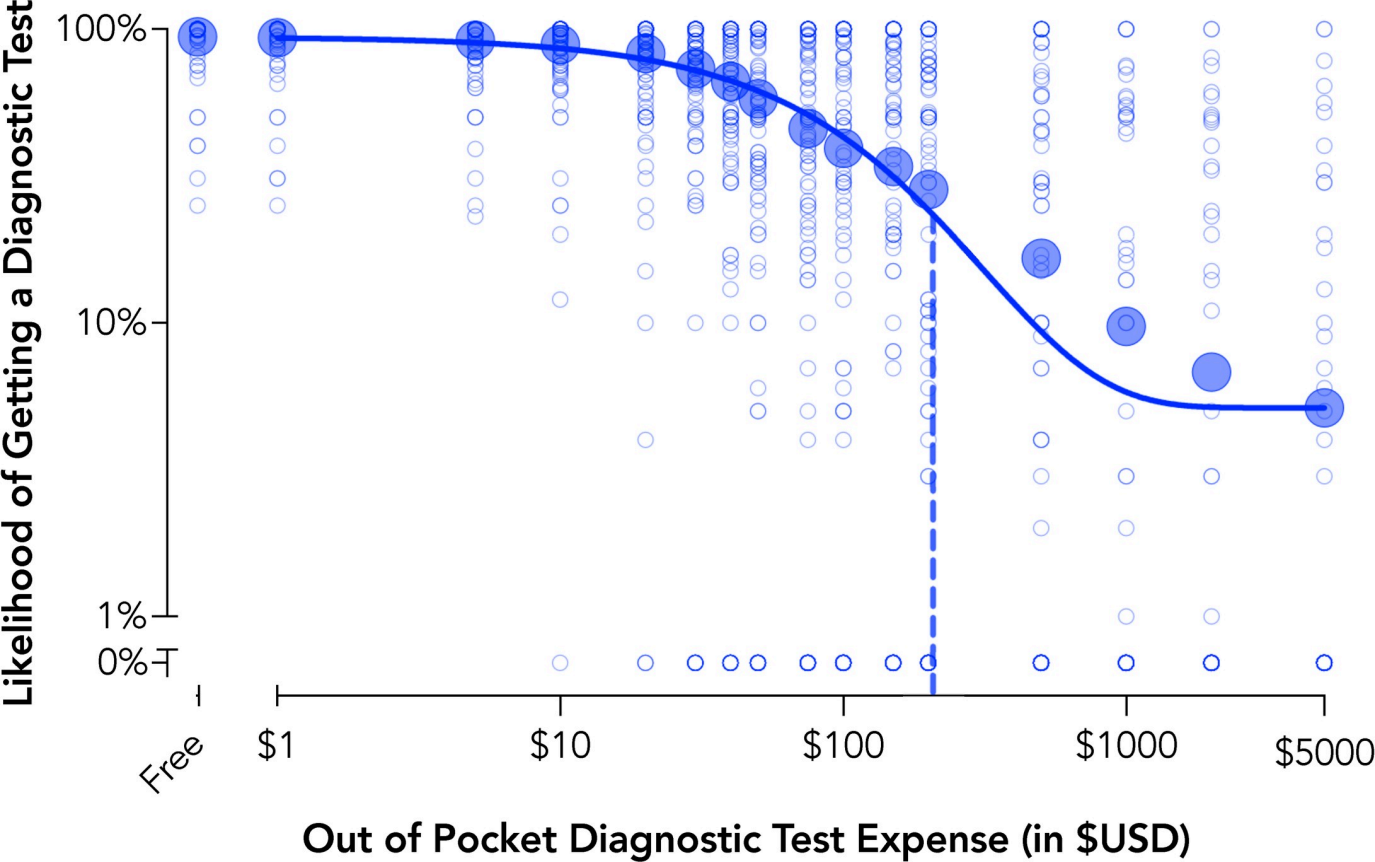
Behavioral economic demand for diagnostic testing (Experiment 4). Plotted are group mean data and individual data points for behavioral economic demand of diagnostic testing recorded on the hypothetical purchase task. Demand curve data are plotted using the exponential demand function [54]. The dotted line is the price representing shifts from inelastic or price insensitive to elasticity or price sensitive demand (P_max_).

Demand for a diagnostic test systematically decreased with increases in cost with the aggregate demand described well, group curve *R*^*2*^ = .98 (Fig 4). The group average demand curve indicated an analytical P_max_ value of $207 indicating the price at which the demand curve shifts from inelastic (i.e., sub-proportional sensitivity of consumption to price) to elastic (i.e., super-proportional sensitivity of consumption to price). Relatedly, the price point of $207 represents the point at which maximal expected revenue from testing is attained (i.e., maximum output or O_max_, an average of $58.37 per customer/participant). Such information may be helpful to inform subsidies, marketing, and commercialization and to provide maximum testing rates and coverage in public health surveillance efforts or ongoing prevalence testing plans.

### Experiment 5 methods

Experiment 5 manipulated the behavioral economic variable of delay to obtaining a diagnostic test as well as the framing of that test (in a choice architecture framework). The primary dependent variable was obtaining a diagnostic test for COVID-19 (dichotomous as explained below). Thus, Experiment 5 measured how the value of a diagnostic test for COVID-19 decreased by the price of the test, delay to obtaining results, and framing of the delay.

Participants completed a delay discounting procedure in which decisions to obtain testing were assessed across systematically varied delays (15 minutes to 28 days). We evaluated two within-subject manipulations in a factorial design. First, *cost* was manipulated with a test as Free or $125 in out-of-pocket expenses (based on the distribution of out-of-pocket costs for COVID-19 testing at the time of the study). Second, *delay framing* was manipulated. In a “Results Delay” condition, participants were instructed that they would receive a test immediately but would have to wait for the test results. In a “Test Delay” condition, participants were instructed that they would receive the results immediately but would have to wait for the test to arrive. Delays were held consistent across these two delay types such that the only stated differences were in the framing of the delay (i.e., as a delay to test result or delay to receiving the test). Participants completed the “Test Delay” condition prior to the “Results Delay” condition with price randomized within these two conditions. Participants were asked if they would get a testing kit given a series of delays (15 min, 60 min, 1 day, 2 days, 3 days, 5 days, 7 days, 14 days, and 28 days). Response options for Experiments 5 as well as Experiments 6 and 7 were simplified as dichotomous yes/no choices rather than the VAS used in prior tasks. This design feature was selected to streamline responding and better model actual decision-making in which decisions are a discrete yes or no choice.

Group data were modeled as in Experiment 1 for descriptive purposes. Maximum delay tolerated for each condition was used as a within-subject, individual subject measure and calculated as the individual median value between last accepted and first rejected delay. Higher maximum delay values are indicative of acceptance of longer imposed delays. Maximum delay values were analyzed using a 2 x 2 repeated measures ANOVA with the within-subject factors of risk Price (Free versus $125) and Delay Framing (Delay to Test versus Delay to Result).

### Experiment 5 results: Delay to test versus delay to result in COVID-19 testing

**Fig 5:**
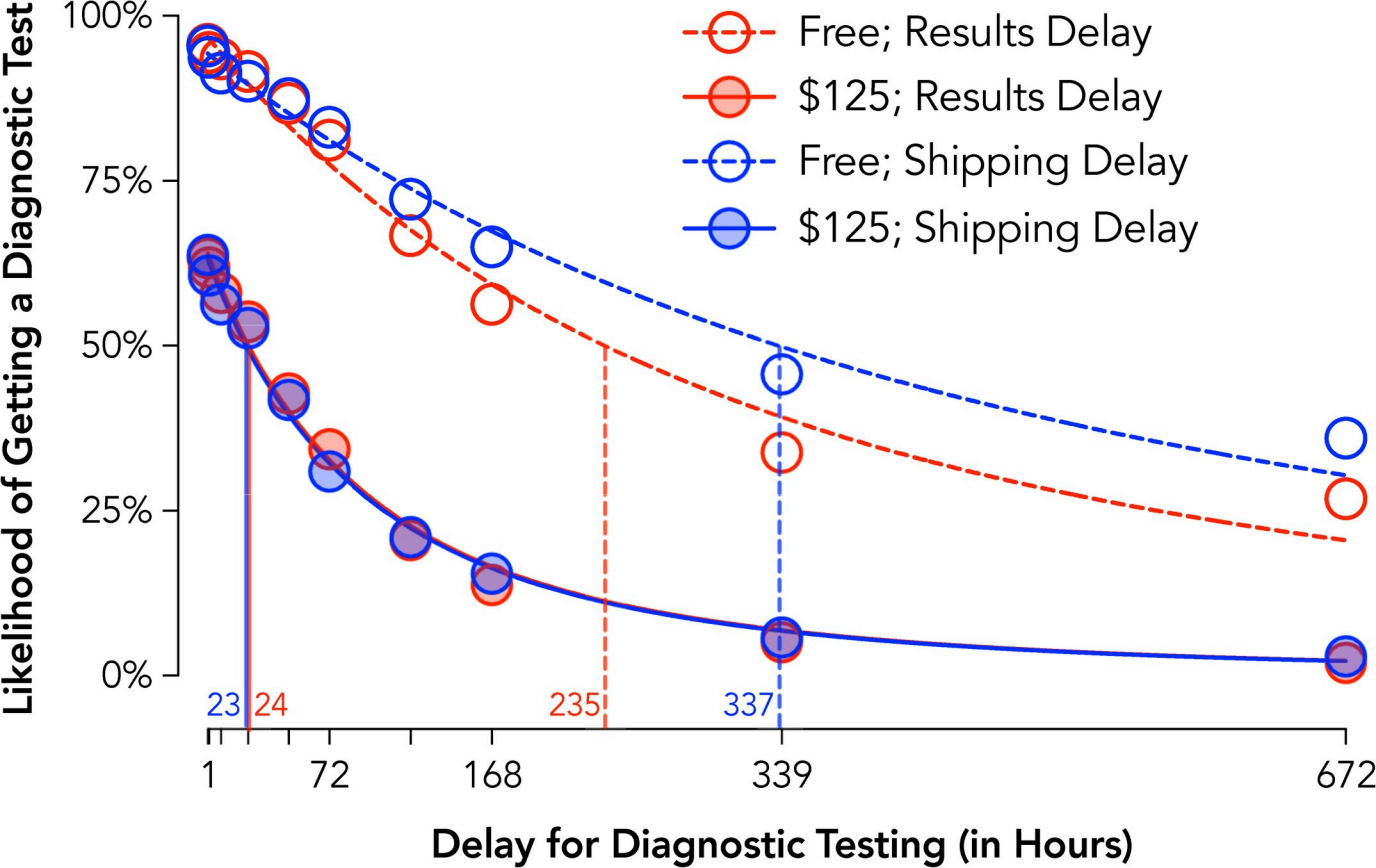
Delay discounting of COVID-19 diagnostic testing by delay type and cost (Experiment 5). Plotted are group discounting curves by delay type (delay to receiving a test with immediate feedback = red circles; delay to receiving results from an immediate test = blue circles) and cost (Free = open circles, dotted lines; $125; closed circles, solid lines). Curves are plotted using the hyperbolic discounting equation including a non-linear scaling parameter [43]. Vertical lines are estimated ED50 or the delay at which half of the population is likely to procure a test.

Assessment of aggregate discounting curves showed systematic reductions in testing intentions with increases in delay for each condition, group curves Free Results Delay *R*^*2*^ = .98, $125 Results Delay *R*^*2*^ = .99, Free Shipping Delay *R*^*2*^ = .98, $125 Shipping Delay *R*^*2*^ = .99 (Fig 5). Tests of individual subject values (crossover delay from “yes” to “no” testing intention) found significant mains effects of Price, *F*_*1*_,_*413*_ = 523.8, *p* < .001, and Delay Framing, *F*_*1*_,_*413*_ = 23.1, *p* < .001, and a Price x Delay Framing interaction, *F*_*1*_,_*413*_ = 30.4, *p* < .001. Evaluation of this interaction indicated that longer delays were tolerated when the delay was to receive a test rather than receive results when tests were free, *t*_*413*_ = 5.64, *p* < .001, *d*_*z*_ = 0.28, but that there were no significant differences by framing when tests carried out-of-pocket costs, *t*_*413*_ = 0.73, *p* = .47, *d*_*z*_ = 0.04.

These findings are highlighted in differences for ED50 values (Fig 5 vertical lines) summarizing the delay at which half the population is likely to procure a test. Specifically, when participants had to pay $125 for test, ED50s of approximately 1 day (23 and 24 hours) were observed in both framing conditions. In contrast, when free, the ED50 was 4.25 days longer (102 hours) for the shipping delay than results delay condition with lower sensitivity to delay in the shipping delay condition.

### Diagnostic testing summary

Experiments 4 and 5 collectively show that the value of obtaining diagnostic tests (and therefore adhering to public health recommendations) were devalued by the manipulated behavioral economic variables of cost and delay. The findings highlight these two behavioral economic mechanisms by which adherence to testing guidelines are impacted, specifically against the price of the test and the delay to its results. These data emphasize how delays imposed on receiving test results may exert a particularly strong impact for discouraging testing, emphasizing how rapid testing may improve testing rates even if a delay is imposed on getting the test. That responding was more sensitive to delayed results than delayed testing is possibly explained by a dominant response (i.e., getting a test) outcome (i.e., receiving a result) contingency at play and how delays for this response-outcome contingency are exaggerated under a delayed results scenario. More broadly, knowledge of what delays are more (or less) tolerable provides a guide as to how to design incentives or subsidies that fall within those boundaries. For example, these findings suggest that if testing is constrained to delayed results, additional incentives may be needed to encourage testing given the greater discounting of testing likelihood observed.

## Vaccination intentions

Recent approval of vaccines for COVID-19 and attempts at distribution have highlighted challenges related to vaccine skepticism and the role of behavior change and motivation as key steps for encouraging vaccine uptake. In Experiment 6, we manipulate the behavioral economic independent variable of probability (vaccine efficacy) to test its impact on COVID-19 and influenza vaccination intentions under a choice framing manipulation (opt-in versus opt-out bundling). In Experiment 7, we again manipulate probability through vaccine efficacy and test the interaction of two choice framing conditions (i.e., safety framing and developmental timeline).

### Experiment 6 methods

Experiment 6 manipulated the behavioral economic variable of probability via vaccine efficacy for a COVID-19 and influenza vaccine under opt-in, opt-out, and no default framing conditions. The primary dependent variable was intentions to receive a vaccination. Thus, Experiment 6 measured how the value of vaccination decreased by vaccine efficacy as well as the impact of a choice framing manipulation.

Participants read vignettes describing a scenario in which approved influenza and COVID-19 vaccines were available. The instructions indicated these vaccines would be the only ones available, that they would be free of cost, would have to be administered now, and were approved by the FDA. Scenarios were presented to simulate going to a healthcare provider for one vaccine and having an option to bundle another vaccine at that visit. Participants responded across a series of efficacies defined as percentage reduction in influenza/COVID-19 symptom risk (100% to 0% effective in 10% increments). Participants were randomized to complete different *choice framing* conditions (between-subject). In an opt-in condition, the response option was preselected as “No” and participants were required to change the selection to “Yes” if they wanted the vaccine (n = 245). In an opt-out condition, the response option was preselected at “Yes” and participants were required to change the selection to “No” if they wanted the vaccine (n = 252). All participants also completed a version in which no response was preselected and were randomized to complete this before or after the choice framed condition.

Group data were modeled using the hyperbolic discounting model as in Experiment 1. An alternative method tested using a behavioral economic demand framework for Experiments 6 and 7 is included in S3 Text. Individual values for minimum required efficacy for each vaccine task were calculated as the individual median value between last accepted and first rejected vaccine efficacy. Participants who rejected the vaccine at all values were assigned a value of 100 and those accepting at all values were assigned a value of 0. Higher minimum required efficacy values are indicative of a need for higher vaccine efficacy for vaccine intention. Minimum required efficacy were first analyzed using a 2 x 2 x 2 mixed ANOVA with the within-subject factors of risk Vaccine Type (COVID-19 and Influenza), Response Type (Default versus No Default) and the between-subject factor of Framing Condition (Opt-In versus Opt-Out). A secondary analysis was conducted with only the first framing condition completed as a 2 x 3 mixed ANOVA with the within-subject factor of Vaccine Type (COVID-19 versus Influenza) and Response Condition (No Default, Opt-In, and Opt-Out).

### Experiment 6 results: Opt-In and Opt-Out procedures for COVID-19 and influenza vaccine bundles

**Fig 6:**
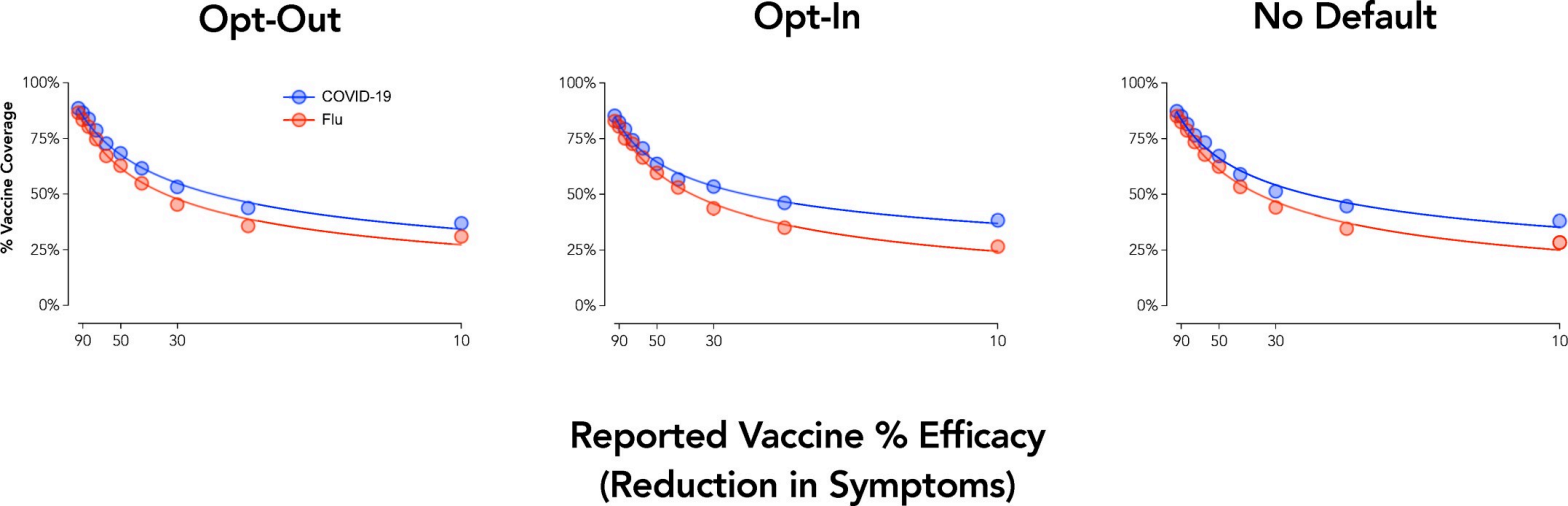
Vaccine acceptance by efficacy, type, and choice framing. Plotted are group discounting curves by vaccine type (COVID-19 = blue; flu = red). X axis values are computed as odds against symptom reduction. Curves are plotted using the hyperbolic discounting equation including a non-linear scaling parameter [43].

Group curves showed systematic decreases in vaccine coverage with decreases in efficacy (Fig 6). The discounting equation described aggregate responding well across each curve, all group curves *R*^*2*^ = .99. Analysis of individual cross-over efficacies (i.e., the efficacy at which a participant went from intending to not intending vaccination) revealed a significant main effect of Vaccine Type, *F*_1,495_ = 39.3, *p* < .001, reflecting vaccine acceptance at lower efficacies for a COVID-19 vaccine than an influenza vaccine. Main effects and interactions involving the framing condition were not significant, *p* > .10.

### Experiment 7 methods

Experiment 7 again manipulated the behavioral economic variable of probability via vaccine efficacy under development timeline and safety framing conditions. The primary dependent variable was intentions to receive a COVID-19 vaccination. Thus, Experiment 7 measured how the value of vaccination decreased by vaccine efficacy as well as the impact of two choice framing manipulations.

Experiment 7 was conducted with a preregistration (https://osf.io/56f2z). Participants completed demand tasks in which we varied *development timeline* (within-subject) as either a 7-month (for late October 2020 delivery) or 12-month (for late March 2021 delivery) process to simulate scenarios presented in news media at the time of data collection (September 2020). Participants were randomized to a *safety framing* condition (between-subject) in which safety was described using a positive framing (“95% of the scientific community declares the vaccine safe”; n = 161) or a negative framing (“5% of the scientific community declares the vaccine unsafe”; n = 161). Assignment was stratified based on endorsement of receiving a flu vaccine in the past three years to ensure balance in general vaccination behavior between the two conditions.

Group data were modeled using the hyperbolic discounting model as in Experiment 1 and individual required minimum efficacy calculated as in Experiment 6. Minimum required efficacy data were first analyzed using a 2 x 2 mixed ANOVA with the within-subject factors of risk Development Timeline (7-month versus 12-month) and the between-subject factor of Framing Condition (Positive versus Negative Framing). A secondary analysis was conducted with only the first task completed as the same 2 x 2 ANOVA with Development Timeline as a between-subjects factor. Based on the preregistration plan, a sensitivity analyses was also conducted including the covariates of age, gender, and conservativism (Social and Economic Conservatism Scale) [56]. A deviation from the preregistered analysis plan was made for this sensitivity analyses because education was not collected in the survey, and therefore, not available to include as a covariate. This analysis used a linear mixed effect model including these covariates, the fixed effects of Development Timeline and Framing Condition, and a random intercept term.

### Experiment 7 results: Development timeline and safety framing for COVID-19 vaccination

**Fig 7:**
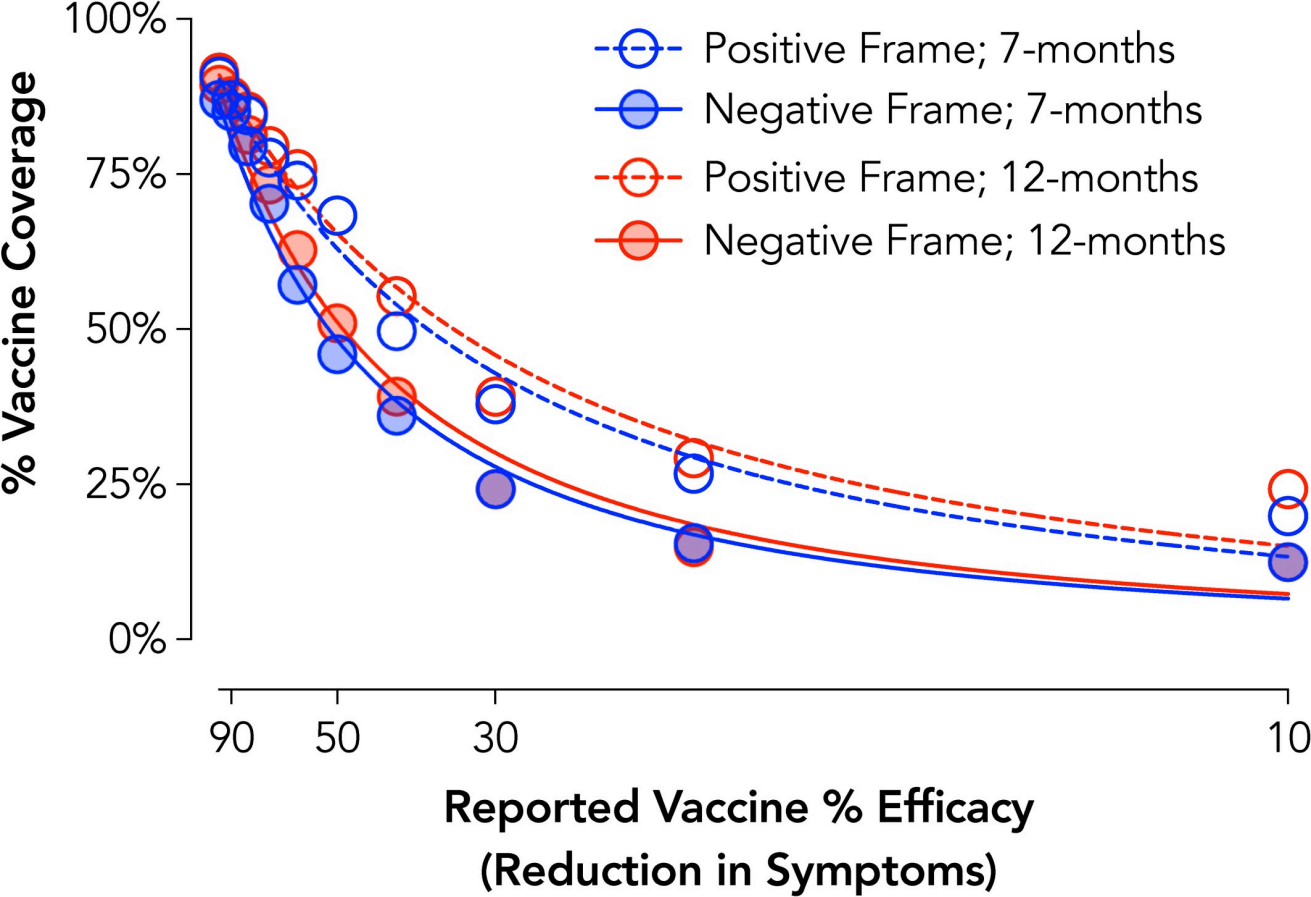
COVID-19 vaccine acceptance by development timeline and safety framing. Plotted are group discounting curves by developmental timeline (7-months = blue; 12-months = red) and safety framing (positive = open circles/dotted lines; negative = full circles/lines). X axis values are computed as odds against symptom reduction. Curves are plotted using the hyperbolic discounting equation including a non-linear scaling parameter [43].

Group curves showed systematic decreases in vaccine coverage with decreases in efficacy across each condition, Positive Frame 7-Months *R*^*2*^ = .98, Negative Frame 7-Months *R*^*2*^ = .98, Positive Frame 12-Months *R*^*2*^ = .97, Negative Frame 12-Months *R*^*2*^ = .98 (Fig 7). At an individual level, significant main effects of Development Timeline, *F*_1,320_ = 9.04, *p* = .003, and Safety Framing, *F*_1,320_ = 14.86, *p* < .001, were observed. These effects reflected acceptance of less effective vaccines under a positive framing condition, *d* = 0.33, and when developed for longer, *d*_*z*_ = 0.22. Controlling for age, gender, and political conservatism did not change the results of these findings. Evaluation of these effects with only the first development time completed (i.e., a purely between-subject design) indicated a similar main effect of Safety Framing, *F*_1,318_ = 7.32, *p* = .007, but found that the Development Timeline effect was no longer significant, *F*_1,318_ = 2.31, *p* = .13. Post-hoc analysis of this possible carryover effect indicated that the Development Timeline effect was statistically significant for participants that completed the 12-month condition first, *t*_*160*_ = 4.77, *p* < .001, *d*_*z*_ = 0.38, but not the 7-month condition first, *t*_*160*_ = 0.73, *p* = .47, *d*_*z*_ = 0.06.

### Vaccination intentions summary

Experiments 6 and 7 collectively show that the value of vaccination for COVID-19 (as well as the flu) was devalued by the manipulated behavioral economic variable of probability (as measured by efficacy). Experiment 6 did not reveal a significant effect of choice framing, which is possibly due to the online setting and limitations of hypothetically simulating opt-in/opt-out procedures. A substantive framing effect for vaccine safety, however, was observed such that intentions were lower under a negative than positive framing. These findings are relevant in that news sources–even when presenting the same data–may focus on either positive (% of scientists approve) or negative (% of scientist disapprove) framings when conveying this information to its readership or viewership [for similar issues in climate change messaging see 57]. The current findings show how such framings informed by behavioral economic theory could adversely impact the likelihood of obtaining a vaccine and ways in which public health messaging should be optimized to avoid such biases.

## General discussion

The COVID-19 pandemic has emphasized how behavioral science is critical to informing public health crisis management. The current study applied the conceptual framework of behavioral economic theory to understand behavioral mechanisms underlying COVID-19 pandemic response. Specifically, we evaluate how behavioral economic variables known to influence other health behaviors of social significance may be adapted to a novel public health issue. Here, we focus on the impact of these manipulated variables like delay to diagnostic testing or probability of community transmission on a larger response class consisting of behaviors underlying public health recommendations to mitigate infectious disease transmission.

The present study advances behavioral science in several ways with each contribution emphasizing behavioral economic theory’s ability to address critical and acute public health crises. First, this study translates operant discounting and demand methods to simulate decision-making in an uncommon context for which a person has no direct experience. The COVID-19 pandemic is a public health crisis, the likes of which have never been experienced by anyone alive today. Although hypothetical discounting and demand tasks are presumed to reflect verbal behavior shaped by histories of consequences in similar choice contexts [58, 59], some decisions lack formal similarity with actual experience. Decisions regarding social isolation, diagnostic testing, or vaccinations for an infectious disease pandemic are relatively novel and require participants to consider generalized decision-making repertoires, such as deciding to take precautions in avoiding people with the common cold or influenza virus. A small, but growing, literature suggests that these kinds of tests of novel or as-yet-unexperienced contexts can nonetheless significantly relate to real-world behavior of interest. For example, in the public health domain, studies on sexual discounting relate to HIV-risk behavior [40, 60] and simulated purchasing of a novel fake ID relate to experienced negative alcohol outcomes in underage drinkers [61]. Moreover, there is evidence that tasks such as hypothetical sexual discounting [62] or hypothetical purchase tasks for drugs [63, 64] significantly predict domain-specific outcomes or behavior beyond general monetary discounting or demand for common commodities. The current study adds to this literature while extending to the study of infectious disease and pandemic response.

As a contribution to the study of infectious disease and pandemic response, the kinds of empirical evaluations reported in this article can reveal new insights related to choice patterns underlying adherence to public health recommendations. We documented the unexpected observation that facemask use was related to social distance, but that people were *less* likely to wear a facemask with those socially close. In this example, we conducted these analyses in advance of the October 14, 2020 statement by the CDC cautioning that small gatherings of friends were responsible for significant spread [65], emphasizing the new insights these kinds of analyses can provide. Such findings, when identified, are especially critical for the formulation of health promotion manipulations and messages. Given that creation of public health messages often focus on expected, rather than observed, patterns of behavior, such novel insights have the potential to better inform messaging to target meaningful choices that would otherwise be ignored.

Second, the data provided by this approach permits safe modeling of potential public health policies. Hursh [14] previously outlined proposed strategies for how behavioral economics can inform health policy, suggesting the quantification of commodity valuation in behavioral economic analyses lend well to informing policy-making. Specifically, experimental research permits controlled and accurate measurement, which may lend new behavioral insights into econometric analyses of market behavior. This information may then inform the creation of experimental model projects to measure scalable policy-level interventions at the community-level. Successful results thereby lead to policy formation, implementation, and evaluation; if there are failures, such results form a feedback loop wherein behavioral scientists can seek to modify procedures and policies to re-evaluate such effects. Related work in psychology and related fields has harnessed hypothetical discounting and demand techniques to provide novel lenses by which to view population-level effects for hard-to-study behavioral questions–from a direct operant perspective, at least–such as tornado warnings [66], incremental cigarette taxation [20], texting-while-driving interventions [67], and happy-hour pricing for alcohol [68]; such findings speak directly to potential population-level decisions and have an added benefit of providing accurate quantitative markers for policy development and targets [14–16].

Finally, this study has potential consequences for understanding behavioral phenomena directly concerning the spread of COVID-19: physical distancing, facemask use, testing procurement, and vaccination intentions. Across several examples, we found that framing manipulations altered sensitivity to the manipulated behavioral economic variables like delay or probability with relevant implications for encouraging (or discouraging) behaviors within this response class of adherence to public health recommendations. Precisely, framing of high-risk social activities increased sensitivity to risk to improve physical distancing, framing delay as a delay to result increased sensitivity to delay for test procurement (thereby discouraging testing), and framing vaccine safety in a negative valence increased sensitivity to efficacy (thereby more steeply reducing vaccine intentions). These framing manipulations can be considered within a broader context of choice architecture manipulations designed to result in subtle, but socially relevant, shifts towards desirable options during health behavior decisions. Prior choice architecture research has shown how similar manipulations can improve influenza vaccination [69] and preventative hand washing behavior [42] (for a meta-analytic summary of framing in health messaging see [70]). The results reported here are consistent with emerging work showing similar impacts of framing manipulations on COVID-19 vaccine intentions (e.g., increased vaccine intentions under personal health risk frames in [71]). The use of simulated discounting and demand tasks in our framing contexts, furthermore, provided a substantive benefit over traditional single discrete-choice forms of assessment (e.g., “Would you get a COVID-19 test?). Such single discrete-choice methods can fail to isolate and control for behavioral economic variables (e.g., differences in delays, risk, efficacy, or safety) that may contribute to observed decision-making. Responding under such methods may therefore be attributable to any of these uncontrolled factors with differing implications for public policy based on the specific mechanism(s) impacted.

These contributions should be considered within the limitations of this study. For one, we restricted sampling to a crowdsourced platform. An extensive body of literature suggests the reliability and validity of data collected through crowdsourced platforms is favorable in comparisons to other convenience methods like undergraduate student pools [72, 73]. Nevertheless, crowdsourcing approaches are still convenience sampling and present some bias such that sampling favors towards younger participants [72]. Crowdsourcing in this context served as an ideal data collection method for generating a large and geographically diverse sample in the face of a rapidly changing public health context in which in-person study was challenging, if not impossible, for this purpose. Some tasks were also evaluated in the same sample of participants as noted for each analysis throughout. A relatively high number of participants displayed non-systematic responding, which may be related to the use of a comparably low prior task approval rate and/or the use of a one-step rather than two-step (i.e., screener and survey) sampling approach [74–76]. Relevant to the specific contributions of these data for COVID-19 and related pandemic responses, our findings are potentially limited by the use of a between-subject manipulation, specific features of the vignette, and collection at a single point in time. Decisions on what was a between- and within-subject manipulation came after careful consideration to maximize a preference for within-subject designs while recognizing design options likely to result in substantive carry-over bias.

These findings are also limited to the hypothetical scenarios used and it is likely that variations of these scenarios would produce further variations in behavior [77–79]. Although the tasks presented were hypothetical in nature, extensive work have found hypothetical versions of these tasks are a reasonable proxy for procedures using real consequences [29–32, 80]. The flexibility of these procedures and ability to evaluate hypothetical decision-making for which incentivized responding is either unpractical or unethical is a major strength insofar as they afford the opportunity to evaluate and compare in short succession a variety of potential contexts relevant to public health response.

Although we adapted the current tasks from previously well-validated experimental procedures, a formal and comprehensive validation process for these modifications was not undertaken (e.g., to evaluate convergent or discriminant validity) and selections relied on face valid selections for task changes. This limitation is offset partly by the observation that the underlying dimensions manipulated (e.g., delay, probability) shifted behavior in a theoretical congruent manner and that many of our framing manipulations similar altered response patterns in an expected direction (e.g., negative framing reduced vaccine intention more rapidly by efficacy). Similarly, we applied traditional analytic methods to evaluate aggregate and individual level responses. Recent advances in the analysis of behavioral economic data such as mixed effect modeling are gaining popularity with theoretical and methodological work preliminarily describing their use [81–83]. Future research may compare these novel approaches to traditional ones to determine their relative utility across broad applied settings. More broadly, the next logical step in this line of work includes the extensive validation of these procedures to determine their applicability in alternative settings (e.g., in-person or clinically relevant populations), utility of variations on these measures (e.g., those with more or less granularity balancing a precision versus time tradeoff), and their predictive validity for long-term clinical outcomes (e.g., prospective prediction of uptake of vaccination).

The COVID-19 pandemic has challenged a spectrum of sciences to reconsider their ability to quickly translate methods to understand, model, and mitigate contagion. The field of behavioral and decision-making science has a rich and productive history addressing issues of societal importance including disease prevention and health promotion. Behavioral economics is a prime aspect of how behavioral science can leverage its methods toward this end, given its ability to address difficult-to-measure behavior and quantify outcomes that are translatable to public health issues. Here we show through a series of key examples across varied COVID-19 behaviors how behavioral economic theory provides a heuristically useful and empirically relevant understanding of behavioral components underlying this global pandemic (COVID-19). Ultimately, these data provide an example of the adaptability and translational utility of behavioral economics when current and future public health crises necessitate behavioral insight and solutions.

## Supporting information

S1 Text Supplemental study methods. (DOCX)

S2 Text Study materials. (DOCX)

S3 Text Experiment 6 and 7 alternative analysis. (DOCX)
