# Supplementary material for: Behavioral economic methods to inform infectious disease response: Prevention, testing, and vaccination in the COVID-19 pandemic
Source: PLoS One. 2022 Jan 19;17(1):e0258828. doi: 10.1371/journal.pone.0258828 (PMC8769299; doi:10.1371/journal.pone.0258828)
Supplement: S1 Text — (DOCX) [file pone.0258828.s001.docx]

**S1 Text: Supplemental Study Methods**

**Sample Characteristics and Systematicity Checks**

*Sample 1 (Experiment 1 and 4)*

Sample 1 was recruited from 13 March 2020 to 17 March 2020. Participants were required to have a 95% or higher approval rate, 100 or more previously approved tasks, and current United States residence to view and complete the study. Compensation for full study completion was $1 USD. A total of 227 participants completed the full assessment.

Data cleaning consisted of evaluation of behavioral economic tasks for systematic responding as well as evaluation of qualitative responses for English language proficiency and comprehension. Probability discounting task (Experiment 1) and behavioral economic demand tasks (Experiment 4) were evaluated using standardized systematic data checks. A total of 31 participants failed checks on the probability discounting procedure, 18 on the demand procedure, and 44 on both procedures. These results closely corresponded to flagged responses on the qualitative data checks with only one additional participant removed based on inattentive qualitative responses. This resulted in an analyzed sample of 133 participants. The analyzed sample was an average of 39.5 years old (SD = 12.1), 59.8% female, and 80.6% White.

*Sample 2 (Experiment 5)*

Sample 2 was recruited from 13 May 2020 to 10 June 2020. Participants were required to have a 95% or higher approval rate, 100 or more previously approved tasks, and current United States residence to view and complete the study. Compensation for full study completion was $1 USD. A total of 499 participants completed the full assessment.

Data cleaning included evaluation of the diagnostic test delay discounting task (Experiment 5) for reversals (i.e., reversing from stating “No” they would not get a test to “Yes” they would get a test). Any participant with one or more reversal on any task was removed (i.e., 1 task = 7 participants; 2 tasks = 8 participants; 3 tasks = 9 participants; 4 tasks = 61 participants). This resulted in an analyzed sample of 414 participants. The analyzed sample was an average of 32.6 years old (SD = 10.9), 58.3% female, and 71.5% White.

*Sample 3 (Experiment 2, 3, and 6)*

Sample 3 completed the assessment from 4 August 2020 to 12 August 2020. Participants were initially recruited from mTurk in March 2020 as a part of a longitudinal cohort with repeated assessments throughout the COVID-19 pandemic. Participants were required to have a 97% or higher approval rate, more than 100 previously approved tasks, and current United States residence to enroll in the parent study. Data collection for this project occurred in Wave 3 of data collection and included 531 participants who completed the full assessment. Participants were compensated $3.50 USD for completion of this assessment.

Data cleaning included evaluation of physical distancing discounting tasks (Experiment 2) and vaccine demand tasks (Experiment 6) for systematic responding. Discounting tasks were evaluated using standardized criteria and demand tasks were evaluated for reversals from a “No” response. A total of 12 participants failed checks on the discounting procedure, 12 on the demand procedure, and 6 on both procedures. An added attention check was included asking about recent use of a fake drug (“oxypentone”) that an additional 6 participants endorsed. This resulted in an analyzed sample of 497 participants. The analyzed sample was an average of 40.0 years old (SD = 11.4), 56.9% female, and 78.7% White.

Of note, non-systematic data were removed for Experiment 3 (“face mask use) specific to Experiment 3. This was done given the atypical nature of the social discounting task and unexpected pattern of response consistently observed (Results Experiment 3). Of the 497 participants in the Sample 3 analyzed set, 45 showed non-systematic responding on any of the social discounting tasks for face masks for an Experiment 3 analyzed sample of 452 participants.

*Sample 4 (Experiment 7)*

Sample 4 was recruited from 12 September 2020 to 23 September 2020. Participants were required to have a 95% or higher approval rate, 100 or more previously approved tasks, and current United States residence to view and complete the study. Compensation for full study completion was $1 USD. A total of 485 participants completed the full assessment.

Data cleaning included evaluation of the vaccine demand tasks (Experiment 7) for systematic responding. Tasks were evaluated for reversals from a “No” response, which were considered non-systematic (i.e., indicating “No” for intention to get a vaccine and then reversing to “Yes” at a lower efficacy). A total of 163 participants failed checks. This resulted in an analyzed sample of 322 participants. The analyzed sample was an average of 38.8 years old (SD = 11.6), 44.5% female, and 76.7% White.
