# Supplementary material for: Behavioral economic methods to inform infectious disease response: Prevention, testing, and vaccination in the COVID-19 pandemic
Source: PLoS One. 2022 Jan 19;17(1):e0258828. doi: 10.1371/journal.pone.0258828 (PMC8769299; doi:10.1371/journal.pone.0258828)
Supplement: S3 Text — (DOCX) [file pone.0258828.s003.docx]

**S3 Text: Experiment 6 and 7 Alternative Analysis**

**
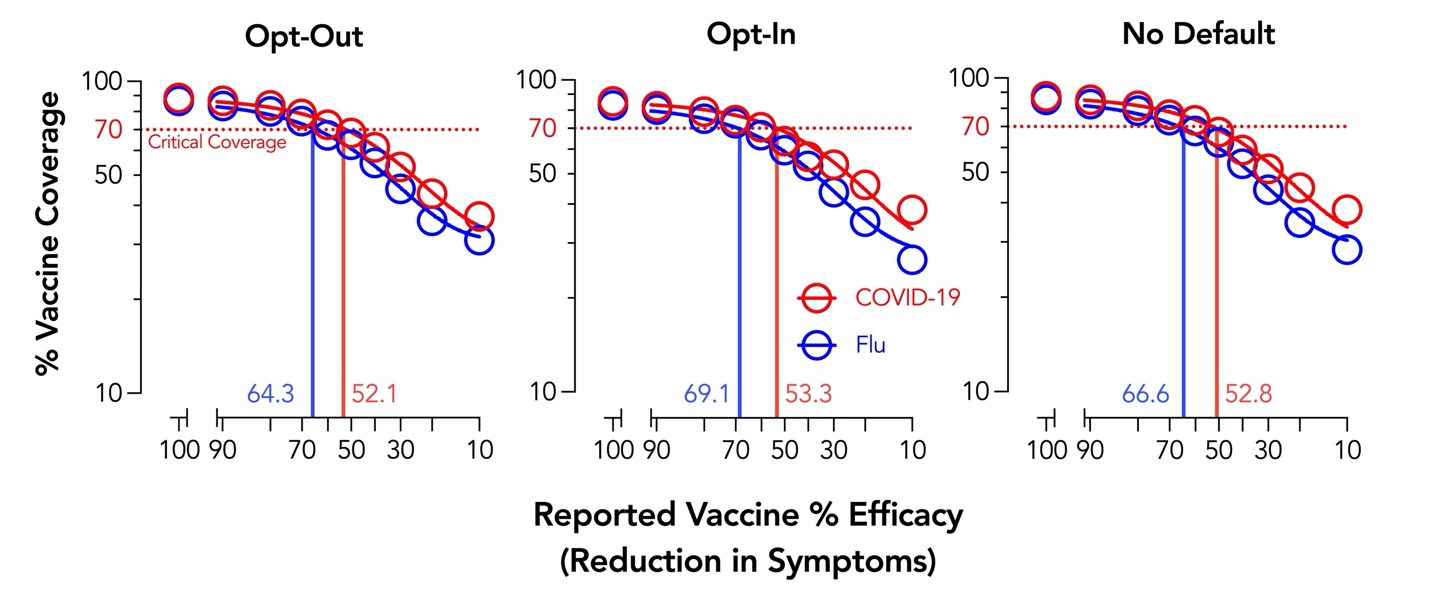
**

**Supplemental Figure 1. Experiment 6 Data Plotted as Demand Curves.** Plotted are group discounting curves by vaccine type (COVID-19 = red; flu = blue). Demand curve data are plotted using the exponential demand function (Hursh & Silberberg, 2008). Vertical lines plot the efficacy needed to reach a critical coverage of 70%.

**
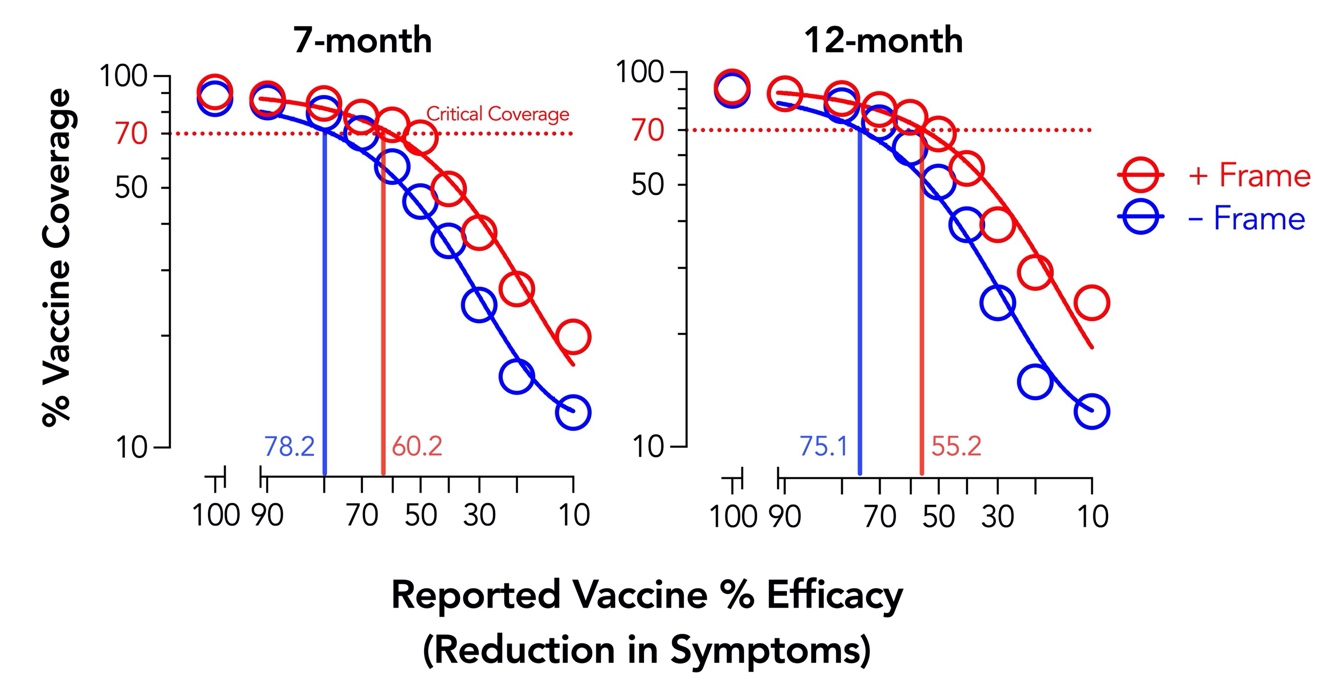
**

**Supplemental Figure 2. Experiment 7 Data Plotted as Demand Curves.** Plotted are group discounting curves by safety framing (positive = red; negative = blue). Demand curve data are plotted using the exponential demand function (Hursh & Silberberg, 2008). Vertical lines plot the efficacy needed to reach a critical coverage of 70%.
